# Green aspects of photocatalysts during corona pandemic: a promising role for the deactivation of COVID-19 virus

**DOI:** 10.1039/d1ra08981a

**Published:** 2022-05-06

**Authors:** Abhinandan Kumar, Vatika Soni, Pardeep Singh, Aftab Aslam Parwaz Khan, Mohammed Nazim, Satyabrata Mohapatra, Vipin Saini, Pankaj Raizada, Chaudhery Mustansar Hussain, Mohamed Shaban, Hadi M. Marwani, Abdullah M. Asiri

**Affiliations:** School of Advanced Chemical Sciences, Shoolini University Solan Himachal Pradesh 173229 India pankajchem1@gmail.com; Center of Excellence for Advanced Materials Research, King Abdulaziz University P. O. Box 80203 Jeddah 21589 Saudi Arabia draapk@gmail.com; Chemistry Department, Faculty of Science, King Abdulaziz University P. O. Box 80203 Jeddah 21589 Saudi Arabia; Department of Chemical Engineering, Kumoh National Institute of Technology 61 Daehak-ro Gumi-si Gyeongbuk-do 39177 Republic of Korea nazimopv@gmail.com; University School of Basic and Applied Sciences, Guru Gobind Singh Indraprastha University Dwarka New Delhi 110078 India; Maharishi Markandeshwar Medical College Kumarhatti Solan Himachal Pradesh 173229 India; Department of Chemistry and Environmental Science, New Jersey Institute of Technology Newark NJ 07102 USA; Department of Physics, Faculty of Science, Beni-Suef University Beni-Suef 62514 Egypt

## Abstract

The selection of a facile, eco-friendly, and effective methodology is the need of the hour for efficient curing of the COVID-19 virus in air, water, and many food products. Recently, semiconductor-based photocatalytic methodologies have provided promising, green, and sustainable approaches to battle against viral activation *via* the oxidative capabilities of various photocatalysts with excellent performance under moderate conditions and negligible by-products generation as well. Considering this, recent advances in photocatalysis for combating the spread of the severe acute respiratory syndrome coronavirus 2 (SARS-CoV-2) are inclusively highlighted. Starting from the origin to the introduction of the coronavirus, the significant potential of photocatalysis against viral prevention and -disinfection is discussed thoroughly. Various photocatalytic material-based systems including metal-oxides, metal-free and advanced 2D materials (MXenes, MOFs and COFs) are systematically examined to understand the mechanistic insights of virus-disinfection in the human body to fight against COVID-19 disease. Also, a roadmap toward sustainable solutions for ongoing COVID-19 contagion is also presented. Finally, the challenges in this field and future perspectives are comprehensively discussed involving the bottlenecks of current photocatalytic systems along with potential recommendations to deal with upcoming pandemic situations in the future.

## Abstract 2

Photocatalysts are green, eco-friendly, clean and sustainable and could be a solution to combat COVID-19 because of potential features of various types of metal oxides against viral inactivation *via* the generation of reactive oxidative species.

## Graphical abstract

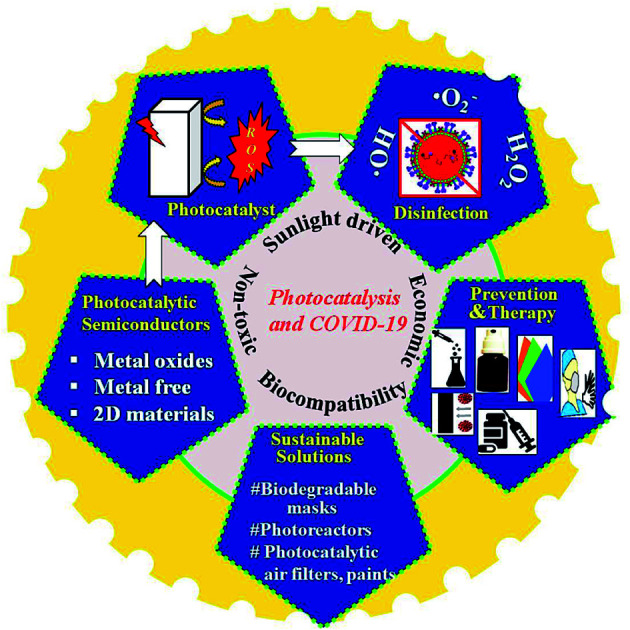

## 1. Introduction

### 1.1 Origin and spread of COVID-19

People with pneumonia who were bordering on acute respiratory patients in Wuhan, China, were first infected with a new, severe acute respiratory syndrome coronavirus (SARS-CoV-2) among different species.^1,2^ After the emergence of coronavirus, the SARS-Cov-2 has been found responsible for infecting millions of people worldwide, which has strong virulence, high spreading nature, as well as contagious nature of the coronavirus (COVID-19).^1–4^ The seventh coronavirus, SARS-CoV-2, is observed to infect humans. MERS-CoV, SARS-CoV-2, HKU1 and NL63 cause severe maladies, whereas OC43 and 229E offer mild and low symptoms.^5,6^ In addition, the SARS-Cov-2 virus acts as an etiologic agent for COVID-19, a global pandemic with no absolute cure according to the World Health Organization (WHO) and COVID-19 infections are still increasing about 250 million confirmed cases and 5 million deaths (on 13 November 2021) in more than 120 countries.^7,8^ Ideally, SARS-CoV-2 is a single-stranded ribonucleic acid (RNA) beta-virus having a non-segmented positive-sense with a spherical shape of 50–150 nm diameter range and club-shaped (S, glycoprotein) spike protein projections.^9,10^ Furthermore, the COVID-19 virus surface is composed of a membrane (M) of glycoproteins and an envelope (E) of the symmetrical nucleocapsid (N) helical with the genome of the virus as well.^10–12^ In addition, SARS-CoV-2 employs entry of cells in the human body with angiotensin-converting enzyme 2 (ACE2) receptor applications.^11–13^ Since, SARS-Cov-2 has been rapidly spreading in symptomatic or asymptomatic patients in different ways as respiratory droplets while talking, skin contacts, air particles or aerosols, and touching contaminated solid surfaces, such as paper, wood, glass, copper, steel, or cloths, and weather conditions, such as light, pH, temperature, or humidity.^14–18^

Owing to the corona pandemic, numerous human societies have to adopt voluntary or government-forced segregation or isolation, use of masks, protective gloves, sanitizer for hand washing, and social distancing by reduced personal contact in public places.^19,20^ Thus, infected or used protective clothes, gloves and masks might have viral traces, which are needed to be separated aside from tens of hours to seven days to self-destroy coronavirus.^21,22^ The scientific methods lack exact coronavirus destruction time, which depends on the components of materials used to manufacture face masks as woven or non-woven polymeric fibers. However, a customer might utilize sanitizing agents for virus protection *via* the interaction between porous fibers and alcohol sanitizers.^21–23^ All types of pathogens, including food, water, and air, might enter *via* different infection modes, resulting in ∼15 million casualties worldwide yearly.^24,25^ Various microbial contaminations, including viral contaminants of drinking water, breathing air, and food products pose huge environmental warnings along with adverse effects on the health of humans.^26,27^ In addition, water-and food-borne outbreaks might be related to environmental conditions with the ecosystem variation all over the world.^28–31^ Clearly, such viruses can be moved by a direct surface contact along with air transfer causing a majorly high risk of the disease compared to other microbial pathogenic contaminants.^32–35^ Due to the growing virus-based epidemic and pandemics in the world, swine flu virus (H1N1), SARS-CoV, MERS-CoV, and novel SARS-CoV-2 (COVID-19) have gained increasing attention of researchers and scientists about viral diseases and their efficient curing.^36–40^

### 1.2 Photocatalysts for COVID-19

**Fig. 1:**
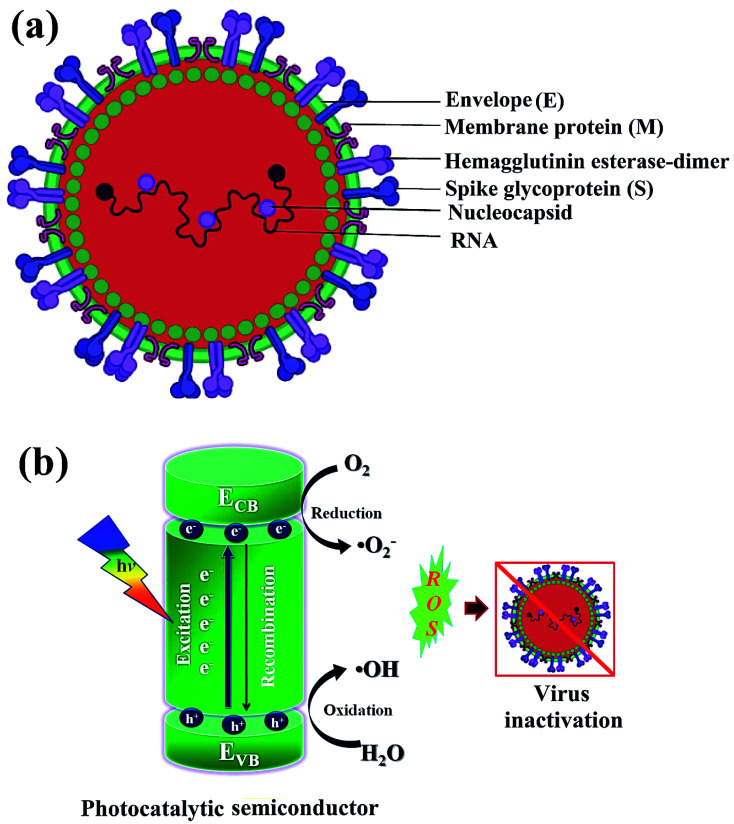
Schematics illustrating (a) structure of SARS-CoV-2 with structural protein and (b) coronavirus inactivation through photocatalysis by the generation of reactive oxidative species (ROS).

Newly identified coronavirus disease (COVID-19) is caused by a highly infectious and rapidly transmitted severe acute respiratory syndrome of coronavirus (SARS-CoV-2).^41^ CoV-2 is a single-stranded (ss) ribose nucleic acid (RNA) genome and was first reported in Wuhan, China in December 2019.^42^ Rapidly transmitted COVID-19 has been declared a pandemic by the world health organization (WHO) since March 2020 in most countries including India, and hence, called the third zoonotic epidemic of the 21st century.^43^ The newly discovered beta SARS-CoV-2 belongs to the family *Coronaviridae*; order *Nidovirales*; genus betacoronavirus that resembled 96.2% with the bat CoV and 79.5% with the spherical SARS-CoV.^44^ SARS-CoV-2 is considered as the largest family of single-stranded enveloped genomic RNA (∼125 nm or 0.125 microns) with 5′-cap and 3′poly-A tail structures.^45^ The functionality of the structural components, including the envelope (E), membrane (M), spike (S), and nucleocapsid (N) are the four major proteins that normalize the function and the structure of CoV-2 (Fig. 1a). Enhanced levels of COVID-19 pandemic and the transmission risk globally are due to the higher estimated reproduction number of SARS-CoV-2 (*R*0, 2–3.5), as compared to SARS-CoV (1.77).^46^

In the last two years, the COVID-19 virus has been intensively researched as extremely communicable where coronavirus efficiently transfers through respiratory droplets and aerosols in terms of cough, spit, or saliva during speaking, sneezing, as well as strong breathing, resulting in millions of cases worldwide. According to the U.S. Centres for Disease Control and Prevention (CDCP, USA) recommendations, personal protective equipment (PPE) including surgical or medical masks, and social distancing are the best and most efficient measures for self-protection from COVID-19 until a reliable or effective solution is obtained to check this coronavirus spread. Among the various types of masks, surgical and N95 respiratory masks are the most effective, which are composed of a thin polypropylene (PP) layer fabric (having negative charge) to filter efficiently particles and droplets containing viruses.^47,48^ Furthermore, various conventional techniques, including chromatography, pasteurization, acidic pH inactivation, precipitation, and UV inactivation are being used to remove virus particles from the wastewater. The use of various chemical methods and exposure of the virus to high temperatures (>65 °C) deactivates the coronavirus to some extent. However, all these conventional techniques are not able to disinfect harmful COVID-19 viruses completely.

Moreover, toxic chemicals or harmful radiations cause etching or allergy in the body when they come in contact with skins and sometimes may evaporate to form secondary contaminants.^46–48^ To overcome such inherited drawbacks associated with conventional methods, many approaches are enlightening different ways to disinfect harmful viruses present on any surface using nanomaterials. In addition, nanomaterials with significant properties invade viruses to enable the expression of antigens; thus, hold great potential to fight against COVID-19. Various nanomaterials having safe, less toxic, economic, and biocompatible properties, are well explored to prevent, detect and disinfect harmful coronaviruses.^43,47–49^

### 1.3 Green aspects of photocatalysis

Green or sustainable chemistry is mainly focused on the design of products and processes that eliminate the use and generation of harmful substances. There is a need to generate antiviral materials that are low-cost, renewable, and easily available to disinfect harmful viruses.^43,49^ In this regard, photocatalysis or artificial photosynthesis is an effective advanced oxidation technology that has received noteworthy attention owing to its ease of handling, low energy requirement and thus, considered as one of the most encouraging technologies for resolving climate-change or environmental pollution problems. Additionally, the provided whole amount of solar energy is more than sufficient, thereby, the real challenge is its effective collection and utilization for living beings.^50^ Of note, photo redox catalysis has several advantages for sustainability; moreover, it also accomplishes several principles of green chemistry.

In photocatalysis, the primary energy source (solar light) is free in an enormous amount, easily available, and environmentally friendly, where in, the absorbed photons provide a huge amount of energy without requiring high temperature or harsh conditions.^51^ The photocatalytic reaction occurs under the joint interaction of light with adequate frequency and the photocatalytic material absorbs a wide range of solar spectra (Fig. 1b). Typically, by acquiring an excited state after light absorption, a single-electron migration event triggers the generation of active electron (e^−^) and hole (h^+^) pairs as excitons in the CB and VB of photoactive semiconductor, respectively.^52^ The isolated e^−^ in the CB can reduce dissolved O_2_ and produce H_2_O_2_, or ˙O_2_^−^ along with H^+^ ions, whereas, the remaining h^+^ in the VB can oxidize the OH^−^ ions from water to generate unstable ˙OH radicals.^53^ These generated reactive oxygen species (ROS; ˙OH, H_2_O_2_, H^+^, and ˙O_2_^−^) participate in the photocatalytic-disinfection and -degradation processes at room temperature.^54,55^ For instance, Matsuura *et al.* analysed a reduced surface spike morphology, increased size, and ∼99.9% antiviral efficiency of the photoactive TiO_2_ against SARS-CoV-2 after 20 min of solar irradiation.^56^ Similarly, Nica *et al.* found iron and nitrogen-doped TiO_2_ nanoparticles as potent antimicrobial candidates that displayed excellent anti-biofilm activity, yet with low toxicity against lung and dermal cells.^55^ Moreover, photoactive antiviral materials with a high surface area were also effectively coated on various textile materials. Furthermore, the virucidal activity of treated fabrics showed improved virus inactivation capacity, while uncoated fabrics showed no activity under light illumination. The hydrophobic interactions were majorly involved in the adsorption of viruses on the surface of coated fabrics, causing distortion of viral shape resulting in induced virus inactivation in ambient conditions. Thus, the surface effect was especially important for TiO_2_ coated samples to observe significant virucidal activity in dark as well. For example, –OH and –COOH functional groups modified TiO_2_ obtained from hydrosol, which significantly facilitated the retention of TiO_2_ particles within the fibers of fabrics. In addition, the presence of hydrosols might increase surface area by one to two orders of magnitude after the addition of TiO_2_ particles. Thus, generated free radicals from TiO_2_ particles might damage the viral surface proteins, which in turn reduce the adsorption capacity of viruses to host cells as well as damage the viral genome, preventing the replication process of the virus.^55,56^ So, the attachment of TiO_2_ particles to cellulose fibers could also be useful to produce virucidal activity in different fabrics. In hospitals, cotton-based items are usually washed and reused because the cotton fabric is covered with TiO_2_ particles, which provides a low level of wash resistance owing to the poor adhesion between TiO_2_ particles and fibers. The high surface area of TiO_2_ particles and hydrophilicity of the coated fabrics could contribute to the antiviral properties. Moreover, similar virus inactivation was observed after one cycle of washing TiO_2_-coated fabrics.^58^ Other potential photocatalytic semiconducting nanomaterials, such as WO_3_, graphene, carbon-based, and 2D nanomaterials are also reported as antiviral agents with long-term photostability under ambient conditions. Hence, heterogeneous photocatalysis using these antiviral semiconductors is relatively safe, non-hazardous and eco-friendly and does not generate any harmful by-products.

During the COVID-19 crisis, metal oxides having self-disinfecting properties were a useful tool for virus protection, interaction and its spread from person to person. In addition, titanium dioxide (TiO_2_) exhibits efficient antiviral, antibacterial, and photocatalytic activities in fighting the COVID-19 pandemic.^57,58^ Under UV light irradiation, the catalytic efficiency of TiO_2_ nanomaterials was diagnosed for virus-deactivation as TiO_2_ nanoparticle thin films deposited on glass, resulting in excellent virucidal performance.^59^ Thus, reactive efficiencies of TiO_2_ nanomaterial surfaces have been efficiently utilized for coronavirus disinfection. Subsequently, cobalt-doped TiO_2_ nanomaterials were reported for the treatment of SARS-CoV-2 infection using a cost-effective electrochemical bio-sensor in nasal or saliva secretions based on spike protein (RBD) sensing on the surface of coronavirus.^60^ Hence, the electrochemical anodization method has been used to fabricate functionalized TiO_2_ nanotubes in the wet-chemical process to precisely detect SARS-CoV-2 in patients in a short time and minimum amounts. Furthermore, TiO_2_ nanomaterials have displayed various benefits including catalytic activity, large surface area, and antiviral applications. Thus, hydrothermally grown photoactive TiO_2_ nanoparticles modify many pathogens of coronavirus at ∼375 nm wavelength under low incident light (∼0.4 mW cm^2^) irradiation^61^ resulting in efficient interaction with the human pathogen, SARS-CoV-2 virus. Here, ROS species formed during light absorption, particularly hydroxyl radicals (˙OH), play a crucial role in attaching and inactivating the SARS-CoV-2 pathogen, which showed huge applicability in medical, chemical and engineering research as well.^60,61^

**Fig. 2:**
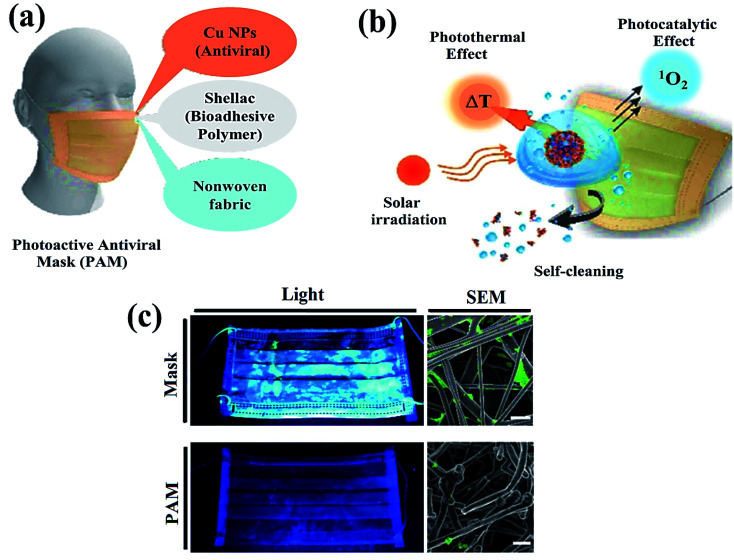
Schematic illustration representing (a) nanocomposite coating and individual components of a surgical mask, (b) viral disinfection *via* photocatalytic, photo-assisted heat treatment, and hydrophobic self-sterilization operations after sunlight illumination, and (c) digital scans (left) and scanning electron microscopy (SEM, right) results of the uncoated surgical mask (top) and photoactive mask (PAM, bottom) with *E. coli* after exposure to solar light for 24 h. Reproduced with permission from ref. 61, copyright American Chemical Society, 2020.

Additionally, photocatalytic antimicrobial materials do not produce any immunological response and are easily compatible when exposed to living tissues. Meanwhile, the mask has been playing a crucial role in controlling the spread of the coronavirus (Fig. 2a) as a point, which displays a clear difference in the SEM images (Fig. 2b and c) of the uncoated and coated masks for virus protection.^61^ For instance, the absence of cytotoxicity after a short time of exposure to the TiO_2_-1% Fe–N-treated cotton knit system, highlighted its potential use in the development of effective antimicrobial agents.^62^ Moreover, photoactive nanomaterials are repeatedly used without any substantial loss in their photocatalytic antiviral activity. In a report, non-toxic photoactive SnO_2_ (core)@ZIF-8 (shell) composite with antiviral properties against chikungunya virus can be effectively reutilized for up to 5 consecutive cycles. In addition, a small amount of photoactive semiconductor material is required to perform effective redox reactions for viral disinfection. Mechanistically, induced oxidative stress (˙O_2_^−^, ˙OH) damaged structural proteins, coenzyme A and disturbed cellular respiration activity of the virus eventually causing cell lysis. Therefore, understanding the real disinfection mechanistic route might help in the development of more powerful photoactive nanomaterials. The green technology thus completely inactivates and degrades species and pollutants without generating any secondary pollution in the environment.^63^

To date, various reports have been filed signifying photocatalytic viral inactivation *via* the generation of a broad spectrum of ROS causing oxidation of organic materials present in viral membranes followed by the destruction of the cell wall and cell rupture, leading to the complete deactivation of the virus. In view of the strong potential of photocatalysis to inactivate various virus species, for the first time, we aim to provide an all-inclusive study that depicts recent advances in the development of distinct photocatalytic materials to prevent and disinfect the SARS-CoV-2 virus. Hence, the green aspects of photocatalysis involving energy sources, types of photocatalytic materials and mechanistic insights are also highlighted and discussed. The significance of photocatalysis involving metal oxides, metal-free, and advanced 2D materials (MXenes, MOFs, and COFs) for antiviral activities is extensively reviewed. Also, a sustainable roadmap to overcome current bottlenecks and promote photocatalytic antiviral performance in order to battle similar forthcoming pandemics is also presented. In conclusion, various challenges in this field and future outlook are deliberated for a better understanding of current limitations and possible solutions to kill this deadly virus.

## 2. Discussion and types of semiconductor photocatalysts

Considering the current emphasis on sustainable energy development, photocatalysis offers a promising alternative for microbial inactivation and prevention with the fascinating features of low to a negligible generation of byproducts, the probable complete deactivation of microbial pathogens and substantial utilization of green solar energy.^64^ In 1994, Sierka and Sjogren's pioneer work represented virus disinfection in water for the first time by using photocatalysis technology.^64^ The study involved a TiO_2_ photocatalyst for the inactivation of MS2 microphages under the illumination of ultraviolet (UV) light.

As a result, the research focus shifted to developing more photocatalytic materials, such as metal oxides, iron-based materials, metal-free photocatalysts, metal–organic frameworks (MOFs), and covalent organic frameworks (COFs) for their potential utilization in antimicrobial activities.^65–72^ Considering this, the following section will summarize various photocatalytic materials and their potential for prevention and disinfection against viral pathogens.

### 2.1 General mechanism of photocatalysis

**Fig. 3:**
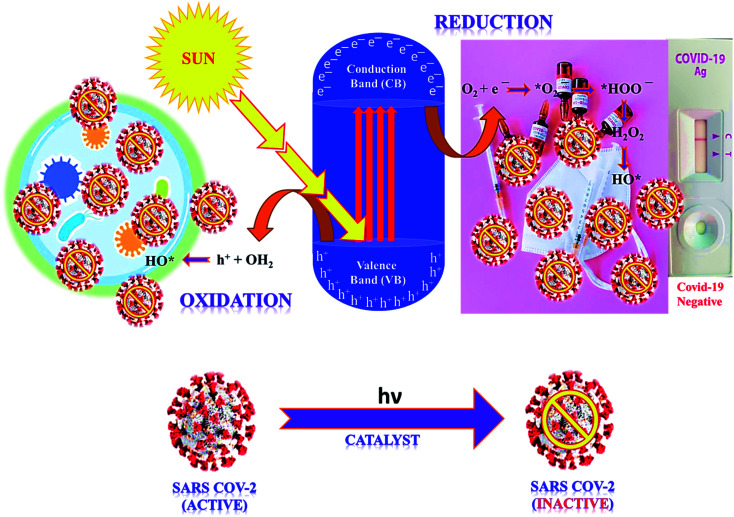
The proposed mechanism of SARS COV-2 virus inactivation in photocatalysis.

In the photocatalysis mechanism (Fig. 3), the photocatalysts excited by ultraviolet/visible light and valence band (VB) electrons move to the conduction band (CB). In the presence of light, the chemical oxidation initiates the disinfection of various types of coronaviruses through short-lived reactive oxygen species (ROS) using catalysts. The virus disinfection can only be obtained with mechanical destruction of the virus after heating or biocidal metal/metal oxide nanomaterials or their composites collectively applied for antimicrobial or catalytic properties.^73^ After light absorption, the formation of ROS efficiently damages the cytoplasmic membrane and cell wall during the virus disinfection mechanism. From VB, the holes interact with ROS species to form an active hydroxyl (˙OH) radical, a powerful oxidant that oxidizes chemicals in the shell and capsid of the virus. Subsequently, the excited e^−^ of the CB interacts with O_2_ to produce hydroperoxide radical (˙OOH) followed by reduced into superoxide radical anion 
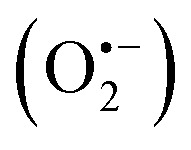
 with the H^+^ ion.^74^ Thus, disinfection of various pathogens might be controlled according to the choice of materials and ROS amount obtained under solar/visible/UV lights. The generated ROS attack interactive sites to control the inactivation of the virus on extra and -intracellular sites.^75^ Furthermore, cell walls and membranes of extracellular active sites have a complicated layered structure with a peptidoglycan layer, a lipopolysaccharide layer followed by phospholipid bilayers. In the cytoplasm of microbial cells, the intracellular active sites are composed of ribosomes, DNA, RNA, and enzymes.^76^ However, the size of catalyst nanomaterials is crucial for efficient catalytic activity and ROS diffusion might be reduced if the size of the catalyst in the cell is more than 300 nm. The cells might be disinfected by oxidation of a non-protein substance, coenzyme A, during the photocatalytic process.^77^ The proposed mechanism of the photocatalytic creation of free radicals follows:

1 Photocatalyst + *hν* → e_CB_^−^ + h_VB_^+^

2 O_2(ads)_ + e_CB_^−^ → ˙O_2_^−^

3 ˙O_2_^−^ + H^+^ → ˙O_2_H

4 ˙O_2_H + ˙O_2_H → O_2_ + O_2_H_2_

5 H_2_O_2_ → ˙OH + OH^−^

6 H_2_O + h_VB_^+^ → ^+^H + OH˙

Thus, light-induced chemical oxidation (Fig. 3) is a promising disinfection method applied in ventilation, air conditioning, and heating to kill pathogenic microbes absorbed on the surface.^78^ However, such dead virus cells gradually reduce their activity with time and are repaired by the doping of catalyst nanomaterials, resulting in an excellent virucidal activity. Thus obtained ROS might impact the lipids, DNA, and cell membranes by the selective attack on nucleotides and sulfhydryl bonds.^79^ During the advanced oxidation process, the biocidal activity of ˙OH radicals might be assessed by its assault on unsaturated fatty acid (FA) chains that result in the peroxidation of lipids and start catalytic chain reactions to damage the cell membrane and FA chains of the viral pathogens.^80^ Thus, formed peroxyl (ROO˙) radicals have low reactivity, and a long half-life to inhibit FA chains than ˙OH radicals that create reactive intermediates. Thus, ROS supports cell damage in oxidative stress, which might be repaired *via* the antioxidant process in microbial cells, where in, microbes might inactivate ROS radicals and destruct healthy cells.^81^ Such ROS collapse different polypeptide chains of proteins by charge modification, resulting in structural modification of amino acids which inhibit the activity of protein due to less active site.

Additionally, ROS, particularly ˙OH radicals, have the ability to change the surface morphology of microbes, including *Bacillus subtilis*, *Bacillus* spores, and *Serratia marcescens*.^82^ Among them, *Bacillus* spore cells contain a hardcover of many layers, which breaks with ˙OH radicals interaction to change its spherical shape and death of cells. However, many microorganisms have the capability to override the oxidation using the superoxide dismutase (SOD) enzyme of the metalloenzyme (MnSOD, Cu/ZnSOD, and FeSOD) family, which degrades the power of oxidants.^83^ In general, *E. coli* produces MnSOD enzymes upon oxygen exposure, which is supported by superoxide radicals. In a similar way, *Staphylococcus aureus* protects ROS through various oxidative-defense methods based on the identification of molecular sentinels with a reply to oxidative stress signals.^84^ While, viruses lacking envelops show less damage, the enveloped viruses display more damage under hygroscopic environmental conditions.

### 2.2 Metal/metal oxide photocatalysts

**Fig. 4:**
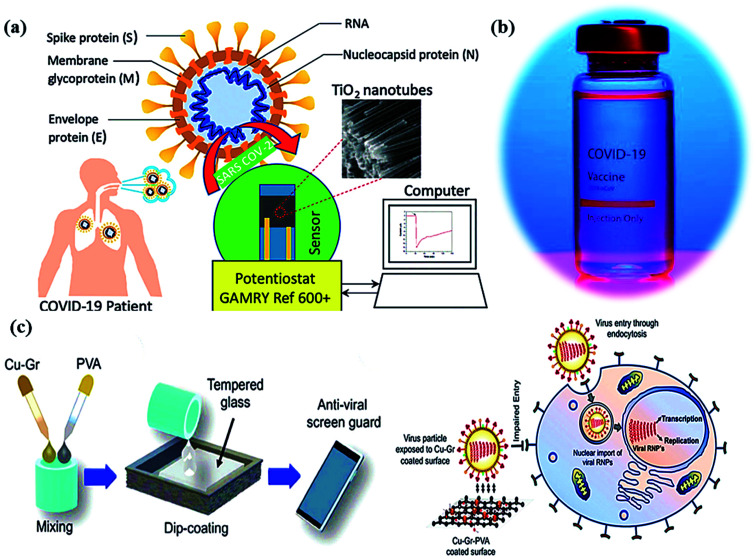
(a) Schematic of a Co-functionalized TiO_2_ nanotube (Co-TNT) based sensing platform for the detection of SARS-CoV-2. Reproduced from ref. 90 with permission from Multidisciplinary Digital Publishing Institute (MDPI), copyright 2020, (b) corona vaccine, and (c) an illustration diagrammatically illustrating the dip-coating technique for tempered glass units with PVA-based Cu–Gr nanocomposite substrates and the mechanism of action for tempered glass surfaces coated with Cu–Gr. Reproduced from ref. 93 with permission from the American Chemical Society, copyright 2020.

Metal oxide photocatalysts offer vital potential to generate ROS under the illumination of solar light and can substantially facilitate photocatalytic viral disinfection. Typically, the generation of ROS involving ˙O_2_^−^, H_2_O_2_ and ˙OH radicals at the surface of photocatalyst is a major step responsible for the disinfection of viruses, due to their ability to oxidize various organic components of microorganisms such as lipid peroxidation resulting in cell wall and cell membrane rupture, protein alteration, and DNA destruction.^85^ So far, TiO_2_ nanomaterials (Fig. 4) are the most explored photocatalyst for microbial disinfection due to their considerably inert behavior, low noxiousness, and significant photostability.^86^ Interestingly, TiO_2_ is widely utilized in paints, self-sterilization windows, lacquers, photocatalytic water purification, and H_2_ generation.^87,88^ Moreover, TiO_2_-containing paints have shown a potential role in ambient air purification due to the ability of TiO_2_ to decompose volatile organic compounds (VOCs) under UV light illumination.^89^ However, the release of toxic products after the degradation of VOCs by TiO_2_-based photocatalytic paints makes this process challenging and questionable.^87^ In that regard, TiO_2_-doped paints could be of utmost interest for surface decontamination and deactivation of SARS-CoV-2 along with aerosol abatement through air filtration filters equipped with TiO_2_ photocatalysts that can be subjected to UV light irradiation.^89^

In molecular docking study, the different iron oxides as Fe_2_O_3_ and Fe_3_O_4_ are shown to interact with glycoproteins of SARS-CoV-2 necessary to bind with receptors of host cells that inactivate the virus by modifying its glycoproteins.^90,91^ Generally, COVID-19 can be measured using a reverse transcription-polymerase chain reaction (RT-PCR) test in the laboratory within ∼2 hours. Thus, a research team has developed a portable device (as nano-PCR) for quick testing within <20 minutes by using plasmonic heating through magneto-plasmonic nanomaterials.^92^ This nano-PCR device (Fig. 4a–c) is reliable, portable, and precise (∼500%), with high sensitivity (∼500%) and excellent specificity (∼500%). It gives on-site COVID-19 detection and provides the establishment of ambulatory clinics for many patients with outstanding accuracy of testing.^93^

**Fig. 5:**
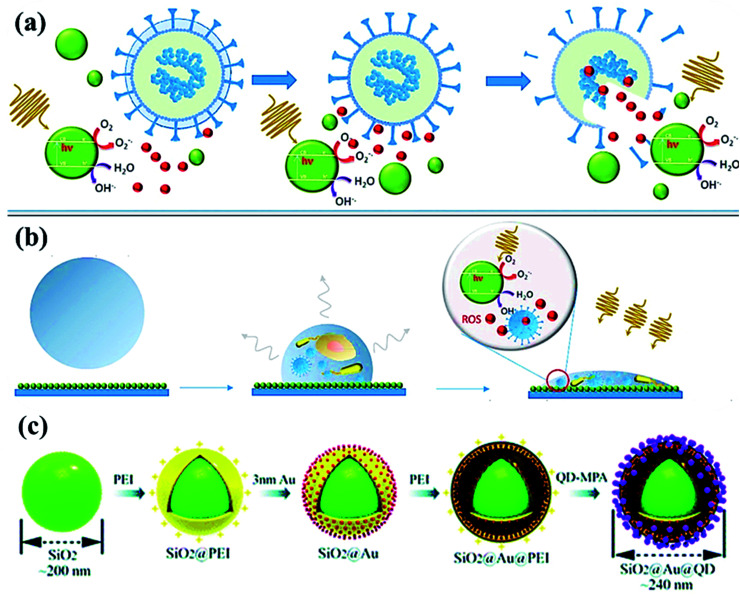
(a) Photoactivation of nanoparticles to produce reactive oxygen species (ROS) during photocatalytic degradation resulting in viral inactivation. (b) Photocatalysis at contaminated NPs coated solid surface upon irradiation, and (c) sequential process for fabricating dual-mode SiO_2_@Au@QD fluorescent labels. Reproduced with permission from ref. (*Current Opinion in Chemical Engineering*, 2019, **1**:100716) from Elsevier, 2019 and ref. 98 of the American Chemical Society, copyright 2020.

The influenza virus is arbitrated *via* endocytosis with viral ribonucleoproteins (RNPs) imported into the nuclei of the host cells. The RNPs accomplish transcription, and replication of the virus in the nucleus to form novel RNPs.^94^ Another potential strategy to prevent viral spread and inactivate microorganisms is by developing photocatalytic antiviral face masks. Owing to hydrophobic features (Fig. 5a and b) and photocatalytic activeness of the coatings, vast research efforts have been put forward to exploit the self-cleaning properties of nanomaterials *via* photo-activation, which can be substantially beneficial for commercial and the medical sector.^95–97^ The surface coating of both Cu and CuO nanoparticles (Fig. 2) has been shown to possess considerable antimicrobial activity even against SARS-CoV-2 after 4 h of exposure to plastics and stainless steel, where the virus can live up to 72 h. Inspired by this, Kumar *et al.* reported a smart and facile technique to construct an antiviral mask by a coating a non-woven surgical mask with shellac/copper (Cu and CuO) nanoparticles to enhance the hydrophobicity of the mask's surface and make it resistant to aqueous droplets (Fig. 2a and b).^60^ The resulting photocatalytic mask exhibited remarkable photo activity and photothermal features for viral activity along with superior reusability and self-sterilization efficacy. As can be seen from Fig. 2c, under the illumination of solar light, PAM suppressed bacterial growth as compared to pristine masks, which is confirmed from SEM images. Under the exposure to light, the temperature at the surface of the mask increased up to 70 °C, leading to the generation of reactive radical species, which facilitated the membrane rupture of nanosized (∼100 nm) viruses.

Recently, SARS-CoV-2-based antibodies might be distinguished by quick, novel and accurate techniques using SiO_2_@Au@quantum-dot (QD) with spike proteins as a lateral flow immunoassay bio-sensor *via* fluorescent detection.^98^ By using serum (∼1 μL) sample, SiO_2_@Au@QD assay exhibits ∼100 times higher efficiency in a short time (<20 min) compared to Au-based assay.^99^ From Fig. 5, the assay developed in the layer-by-layer assembly of SiO_2_ nanosphere followed by the polymer [poly (ethylene imine), PEI] with the positive-charge formation on the surface. In addition, negatively-charged Au nanoparticles (Fig. 5c) of <5 nm size were formed followed by the PEI polymer coating, then, functionalized-quantum dots (carboxylate QDs) layer, which created a strong fluorescent colorimetric signal during sensing.^98–100^ Meanwhile, SARS-CoV-2 virus spike protein forms covalent-coupling in SiO_2_@Au@QDs (Fig. 5c) followed by deposition of anti-human IgM/IgG on the test lines to obtain efficient, selective and COVID-19 virus-sensitive reports.

### 2.3 Metal-free photocatalysts

**Fig. 6:**
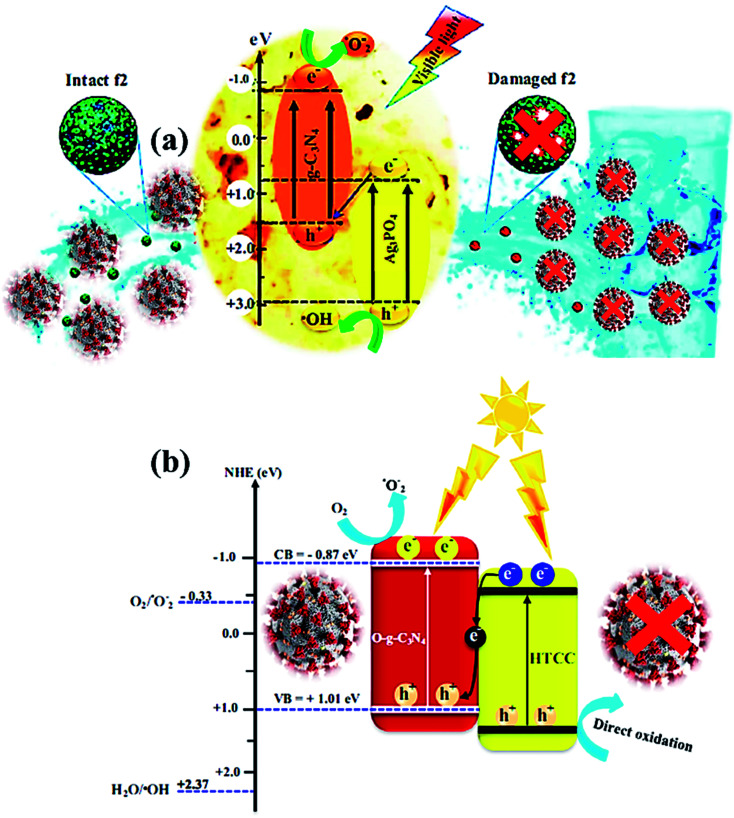
(a) Photocatalytic mechanism for bacteriophage f2 inactivation by AgCN photocatalysts under visible light. Reproduced with permission from ref. 108 copyright 2018 Elsevier, (b) possible Z-scheme mechanism for O-g-C_3_N_4_/HTCC photocatalyst. Reproduced with permission from ref. 110 copyright 2019 Elsevier.

Visible light active metal-free photocatalysts represent a promising category of cost-effective, non-toxic, and stable semiconductor materials, which should be substantially explored for energy generation and water treatment applications.^101–105^ Although metal-free photocatalysts (Fig. 6) such as graphene, graphene oxide, and g-C_3_N_4_ have not much exploited for fighting SARS-CoV-2 infection yet, their fascinating physicochemical and antiviral activities recommend the potential usage of these materials in preventing and disinfecting COVID-19 viruses *via* the construction of coated air filters, face-masks and waste-water disinfectants. Typically, the antimicrobial activity of graphene nanomaterials relies on various effects such as membrane stress, oxidative stress, and photothermal stress along with charge migration and entrapment influence of graphene derivatives.^106^ However, in contrast with the bactericidal activity, the antiviral activity of graphene-based nanomaterials is less studied mainly due to the difference in the size of virus (2–300 nm) and bacteria (500–5000 nm) making analysis problematic and expensive. Akhavan *et al.* investigated the antiviral activity of graphene–tungsten oxide nanocomposite for photo-assisted inactivation of bacteriophage MS2 viruses.^106,107^ For understanding mechanistic insights into the deactivation process, the destruction of protein capsid and efflux of viral RNA encapsulated in protein was investigated. The results further revealed that graphene–tungsten oxide nanocomposite showed significant recyclability with less than 10% decline in the RNA efflux after 20 catalytic cycles under 60 h irradiation period.

The photocatalytic inactivation was applied using Ag_3_PO_4_/g-C_3_N_4_ nanocomposites (AgCN) in hydrothermal synthesis to study the bacteriophage f2 virus.^108^ Due to the combined effects of Ag_3_PO_4_ and g-C_3_N_4_, the performance was boosted as the proposed photocatalytic Z-scheme mechanism (Fig. 6a) *via* wide absorption of the visible spectrum and efficient charge carrier separation. The inactivation Z-scheme mechanism of the f2 virus by binary nanocomposite catalysts was performed with radical quenching tests, which exhibited catalytic disinfection efficiency of the f2 virus found of ∼6.5 log in 80 min on visible-light exposure due to selective virus damage by the ROS after charge separation through g-C_3_N_4_ and Ag_3_PO_4_ components.^109^ Hence, a novel, promising nanocomposite photocatalyst was developed for viral disinfection originating from contaminated water. In addition, a new metal-free nanocomposite was synthesized in a two-step hydrothermal process as oxygen-doped g-C_3_N_4_/hydrothermal carbonation carbon (O-g-C_3_N_4_/HTCC) microspheres. This nanocomposite displayed excellent virucidal efficiency for HAdV-2 with visible-light absorption to disinfect 5 log in 2 h under optimized conditions. Thus, improved disinfection activity of O-g-C_3_N_4_/HTCC nanocomposite against the virus was governed through the Z-scheme mechanism with efficient OH creation to heavy damage of the rigid capsid of HAdV-2 (Fig. 6b) after an excellent charge separation process.^110,111^

### 2.4 Antiviral activity of GO

**Fig. 7:**
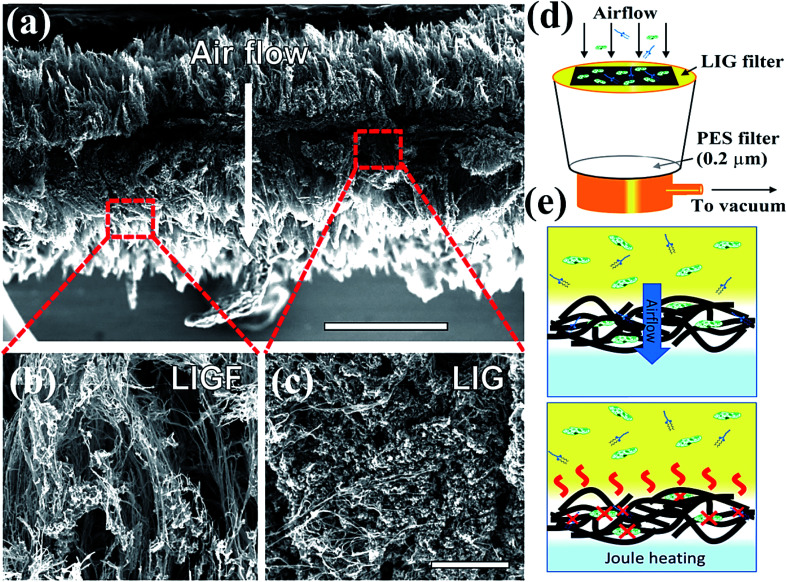
Schematics representing (a) intrinsic morphology and thickness of laser-induced graphene (LIG) filter, which boosts filtration efficacy (b) SEM image depicting the outer fibrous carpet region of the LIG filter useful to trap larger constituents and aerosols, (c) SEM image showing porous graphene portion possessing tortuous 2.86–8.94 nm pores, which facilitate bacterial and finer particle capture, (d) LIG filter system placed on a vacuum filtration arrangement, (e) schematic depiction of filtration (top) leading to sterilization and Joule-heating assisted depyrogenation (bottom). Reprinted with permission from ref. 117, copyright American Chemical Society, 2019.

In general, GO is the oxidized product of graphene with different oxygen-based function groups on the surface as ketone, epoxide, hydroxyl, and carboxyl groups.^112^ GO displays excellent hydrophilicity, bonding ability, and wettability compared to pristine graphene due to the presence of more active sites on the edges and surfaces.^113^ In addition, GO is applicable as an antibacterial and anti-cancer agent owing to its high conductivity, mechanical strength, surface-to-volume ratio, and 2-D monolayer structure with a negatively charged surface. GO might interact with the plasma membrane of viruses to produce reactive oxygen species (ROS) and the antiviral activity of GO was measured with a DNA virus called porcine herpes virus, resulting in the inhibition of virus infection in non-cytotoxic samples.^114^ In addition, GO/PVP nanocomposite has exhibited strong antiviral activity owing to the non-ionic nature of PVP polymer where GO might damage virus structure by reducing virus insertion into host cells.^115^ Graphene oxide (GO) is another potential semiconductor material possessing surface functionalization of hydroxyls, ketones, epoxides, diols, and carboxyl groups, which was found beneficial for rupture of coating proteins leading to efflux of RNA under aqueous conditions.^112–114^ The oxygen atoms present at basal planes render superior hydrophilicity, dispersibility in H_2_O and attachability to GO in contrast with graphene, leading to better antimicrobial activity. However, the interaction of GO with proteins after the inactivation process results in superficial bioreduction of GO into graphene form. On the other hand, the reduced form of GO, called reduced graphene oxide (rGO) functionalized with polysulfated dendritic polyglycerol has also exhibited considerable inactivation features against several viruses encompassing orthopoxviruses, equine herpesvirus type 1 (EHV-1), and herpes simplex virus type 1 (HSV-1).^115,116^ With further advancements in technology, graphene-based materials are widely explored for decontamination and disinfection of SARS-CoV-2 by constructing self-sterilized air filters and antiviral face masks, which are necessary prevention measures against COVID-19. For instance, Stanford *et al.* developed a self-sterilized air filter equipped with laser-induced graphene (LIG), which is microporous and conductive in nature.^116,117^ Typically, a free-standing LIG membrane comprising a carpet of porous fibers (Fig. 7a–c), which promoted the capture of microorganisms, specifically bacteria along with a restriction on the proliferation of filtered microorganisms. Furthermore, LIG membrane filter, as shown in Fig. 7d, was supported with periodic Joule-heating, which boosted the temperature (>300 °C) and facilitated the decomposition of bacteria along with other molecules and microorganisms such as pyrogens, endotoxins, exotoxins, allergens, mycotoxins, prions, and nucleic acids (Fig. 7e). Therefore, by integrating nanosized membrane technology with a greener and advanced photocatalytic process, graphene-based materials could be effectively utilized to prevent and fight against COVID-19.

As a metal-free photocatalyst, g-C_3_N_4_ can be another potential candidate for visible light-enabled photocatalytic inactivation of the SARS-CoV-2 virus. To date, the antiviral and antibacterial activity of g-C_3_N_4_-based photocatalysts has been substantially evaluated owing to its suitable bandgap of 2.7 eV with appropriate valence and conduction band potentials. For instance, g-C_3_N_4_ exhibited superior inactivation of *E. coli*,^118^*Staphylococcus aureus* (*S. aureus*),^119^ MS2 bacteriophages,^120^ human adenoviruses,^121^ and *Bacillus anthracis* endospores^122^ by the generation of reactive radical species under the illumination of UV-visible light.

### 2.5 Other 2D materials

**Fig. 8:**
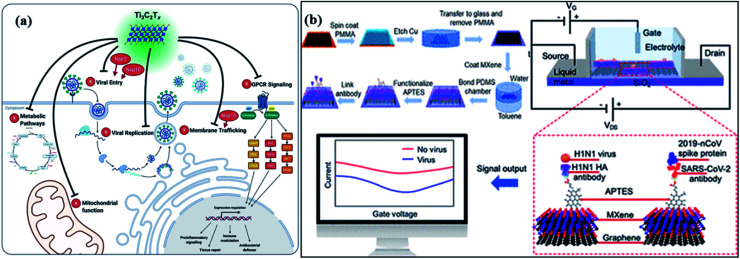
(a) The mechanism of MXene-dependent viral inhibition of Ti_3_C_2_T_*x*_ to explore viral inhibition activity at the cell surface, and (b) an illustration of antibody-antigen sensing in a FET circuit and change in the drain-source current is achieved by MXene–graphene VSTM deposition. Photograph: courtesy of “Yanxiao Li”. Copyright 2020 and the image is of the free domain.

For catalytic deactivation of SARS CoV-2 virus, several 2D materials such as MXenes, metal–organic frameworks (MOFs), and covalent organic frameworks (COFs) are emerging potential semiconductor photocatalysts with alluring features involving good conductivity, layered structure, mechanically firm, flexible, large surface area, and offer high affinity for guest materials.^123,124^ Typically, 2D carbides and nitrides (MXenes) having formula M_*n*+1_X_*n*_T_*x*_ (where M = Ti, Zr, V, Mo, *etc.*, X = C and/or N, and T_*x*_ depicts surface-functionalized =O, –OH, –F, and –Cl groups) with *n* ranging from 1 to 4, offer significantly high surface area and porosity leading to superior adsorption of guest molecules and viruses.^125,126^ Moreover, the visible light activity of these materials facilitates the inactivation of the surface adsorbed virus *via* the generation of ROS. Additionally, the plasmon resonance property of MXenes (Fig. 8) under visible or infrared (IR) light exposure helps to convert light into heat (photothermal effect), which further adds up to deactivate viral species and even enables their application in phototherapy.^127^ Recently, in order to amend the photocatalytic antibacterial activity of MXenes, a Schottky heterojunction with interfacial engineering based on the work function values of Ti_2_C_2_T_*x*_ MXenes coupled with Bi_2_S_3_ was constructed.^128^ The work function engineering helped boost charge carrier transference and enabled the fast killing of bacteria. Other than that, MXenes and their composites can be utilized as protective coatings on the personal protective equipment (PPE) leading to a promising alternative to develop reusable PPEs in order to overcome the burgeoning disposal waste crisis.^127,129,130^

In addition, an MXene-dependent antiviral action mechanism (Fig. 8) has been proposed using proteomics data for docking analysis to compare with SARS-CoV-2 protein interactions. Various SARS-CoV proteins, including NSP7, NSP10 and NSP15, interact with MXenes (as Ti_3_C_2_T_*x*_) to produce obstacles in the viral lifecycle *via* membrane trafficking followed by G-protein coupled receptor (GPCR) signaling, and mitochondrial function for metabolic pathways resulting in viral replication.^131^ Therefore, the capability of MXenes provides an excellent antiviral activity by modulation of viral proteins (Fig. 8a) containing host proteins such as GRPEL1, NUTF2 and GNG5, which regulate antiviral activity. Furthermore, the interaction of host proteins with SARS-CoV (red-colored) proteins. In addition, viral protein, NSP7 is present in membrane trafficking and GPCR signaling while NSP15 is involved in nuclear transport and vesicle trafficking in SARS-CoV-2 infection. Frequently, NSP7 and NSP10 modify endomembrane to support the insertion of a virus and its replication. Hence, the Ti_3_C_2_T_*x*_ treatment functioned in all pathways of Vero E6 cells (Fig. 8b), resulting in SARS-CoV-2 inhibition.^132^

**Fig. 9:**
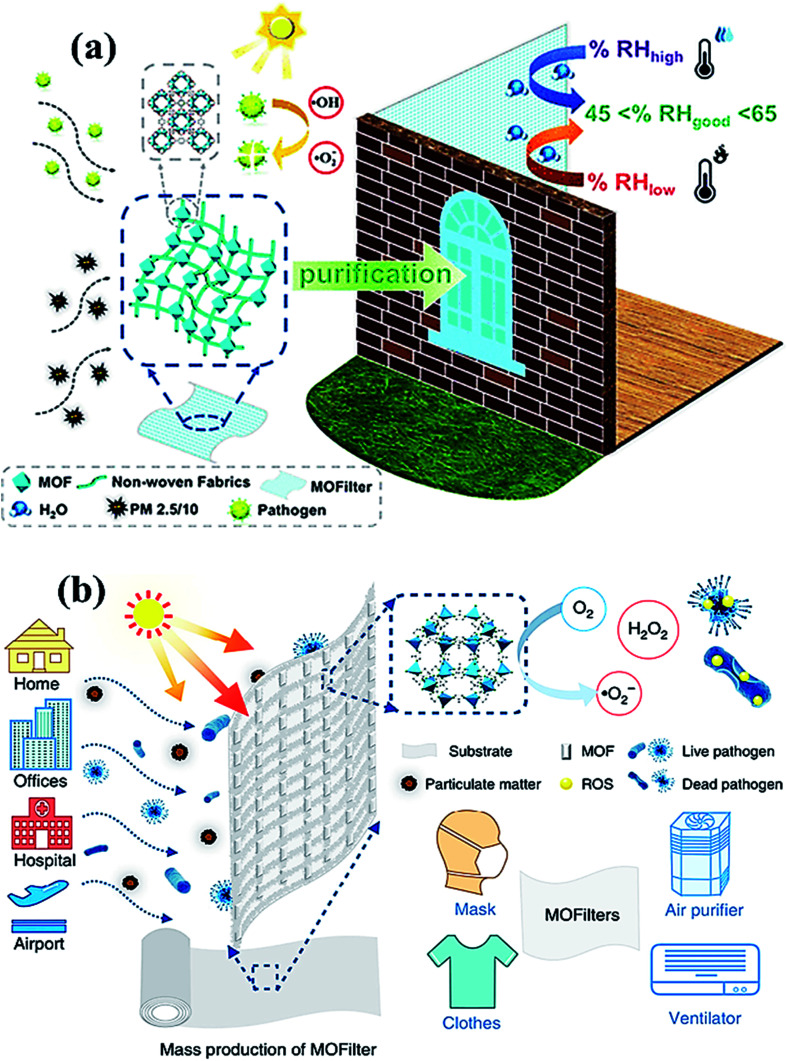
(a) MOF filter (CAU-1) using functionalized terephthalic acid ligands for indoor humidity, and microbial growth, to prevent pollution. Reproduced with permission from ref. 138, copyright American Chemical Society, 2020. And (b) MOF-based filters used in different living areas. Reprinted with permission from ref. 141, copyright © 2019 Springer Nature Limited, Nature, 2019.

Another fascinating class of 2D materials with excellent photocatalytic activities involves metal–organic frameworks (MOFs) and covalent organic frameworks (COFs). The potential features of these materials involve high porosity, physiochemical stability, structural tunability, wide host–guest interactions, sorption, and ion release ability, which make them a potential candidate for various photocatalytic applications and in biomedical fields.^133,134^ Recently, zinc-based imidazole MOFs (ZIF-8) have shown outstanding ∼100% virus-inactivation efficiency in 30 minutes against *E. coli* under solar light owing to their excellent destruction of the cell wall of bacteria. Hence, MOFs (Fig. 9) might be applied in the industrial-size production of filters for air-cleaning masks, cloths, ventilators, and air purifiers.^135^ Recently, bismuth and bismuth–graphene (Bi@graphene) nanocomposites were developed as a photocatalytic air purifier under UV irradiation. Furthermore, Bi@graphene nanocomposites showed excellent photocatalytic deactivation performance against *E. coli* compared to the pristine bismuth nanospheres.^136^ Thus, the improved antibacterial activity of nanocomposites related to highly oxidative ROS generation from Bi surface followed by graphene results in strong excitation and fast charge transfer process.^137^ Subsequently, aluminum-terephthalate-based MOFs (Fig. 9a) have been reported to construct air purifiers for airborne bacterial removal and humidity management for indoor applications. Importantly, monohydroxy terephthalate-based MOFs displayed excellent disinfecting photocatalytic performance of ∼99.94% under <60% RH, and ∼500 cycling performance against *E. coli* bacteria.^138^ Specifically, monohydroxy-terephthalate-coated air filters of non-woven fabric protect efficiently against sudden humidity changes of air in outdoor conditions. Thus, this work gives a meaningful outcome for the next-generation development of antibacterial, water adsorbents, and active filters to manage the quality of air and humidity under indoor conditions.^136–138^

Owing to their tunable structure, biocompatibility, and encapsulation ability, both COFs and MOFs have been utilized as drug delivery agents with optimal efficacy.^139,140^ Nevertheless, to prevent and control the viral spread, MOFs embedded air filters, which possess superior adsorption and photocatalytic virucidal and bactericidal activities have also been explored and reported lately.^141^ Moreover, Li *et al.* reported [Ag_4_(μ-PTA)_2_(μ_3_-PTA)_2_(μ_4_-pma)(H_2_O)_2_]_*n*_·6*n*H_2_O (bioMOF 1) bioactive metal–organic framework (bioMOF 1) and investigated antiviral, antibacterial, and antifungal properties under exposure to light. The report suggested that the bioactive MOF (Fig. 9b) successfully disinfected *E. coli*, *P. aeruginosa* and *S. aureus* bacteria along with *C. albicans* yeast with considerably lower minimum inhibitory concentration (MIC) values. Furthermore, bioMOF 1 also showed HAdV-36 deactivation activity and high cytotoxicity toward abnormal epithelioid cervix carcinoma (HeLa) cell line, suggesting the potential of MOFs against the COVID-19 virus. Therefore, MOFs and COFs could deactivate the SARS-CoV-2 virus by eliminating crown-like spike proteins by perforating the lipid membrane along with the efflux of RNA content for about 3 h under the illumination of UV light. However, the generation of ROS leads to impairment of spike proteins, resulting in superior virucidal activity under the photocatalytic inactivation process.^142,143^

### 2.6 Durability of photocatalysts

To detect viral particles in the air, various photocatalyst-based methods have been applied for virus inactivation during photocatalysis, which efficiently reduces the virus load in the air under indoor conditions. Thus, photocatalysis has been used to effectively remove various pollutants in gas or aqueous phases.^144^ Despite the precise removal of biological pollutants, the photocatalytic viral model might not be useful to different viruses in indoor air applications, such as volatile organic compounds (VOCs), pesticides, and dyes. During photocatalysis, virus inactivation is tuned according to the temperature and humidity of the environment with a change in the rate of photocatalysis. In airborne viruses, the viral inactivation mechanism might proceed through chemical oxidation through toxic metal ions released along with viral surface destruction.^145^ The flower-like TiO_2_ micro-nanoparticles were deposited on a cotton-type fabric surface using the hydrothermal method. The high thickness silver-nanoparticle (Ag NPs) film was formed uniformly on the surface of the TiO_2_@cotton fabric after sodium hydroxide pretreatment followed by *in situ* AgNO_3_ reduction. During the hydrothermal reaction, the concentration of AgNO_3_ has a crucial impact on microorganisms to tune their antibacterial activity along with high UV protective ability, having excellent 56.39 ultraviolet protection factor values.

Thus, the prepared fabric or textiles display excellent mechanical strength and durability after the rough cleaning and abrasion process. The multilayer surface roughness explores its mechanical stability, resulting in longer durability at high pressure of ∼255 kPa during textile, medical, or hygiene applications.^146^ During the COVID-19 pandemic, such fabric might repel coronaviruses, such as SARS-CoV-2 resulting in excellent safety for healthcare workers. Thus, PPE kits or medical clothing could be fabricated from various types of bacteria-, protein, blood, and virus-repelling fabrics in the near future (Table 1).

**Table 1:** Various types of photocatalysts applied for viral disinfection

Photocatalyst	Types of virus	Amount of catalyst (mg L^−1^)	Light source	Time of disinfection	References
TiO_2_	Phage f2	1000	Black light	15 min	78
TiO_2_	*Murine norovirus*	10	UV lamp	24 h	16
Ag–TiO_2_	Hepatitis B	100	UV lamp	12 h	12
*n*Ag/TiO_2_	Phage MS2	100	UV lamp	80 min	76
Cu–TiO_2_	Bacteriophage f2	50	Xe lamp	120 min	47
Mn–TiO_2_	Phage MS2	100	Xe lamp	60 min	26
SiO_2_–TiO_2_	Phage MS2	100	UV lamp	1.8 min	71
C_60_/SiO_2_	Phage MS2	500	Sunlight	75 min	25
C_70_/SiO_2_	Phage MS2	300	Sunlight	90 min	87
g-C_3_N_4_	Phage MS2	135	Xe lamp	240 min	85
O-doped g-C_3_N_4_	Human adenovirus	—	LED lamp	120 min	31
Ag_3_PO_4_/g-C_3_N_4_	Bacteriophage f2	100	UV lamp	80 min	49
Ag–AgI/Al_2_O_3_	Human retrovirus	320	Xe arc lamp	40 min	22
FeO	Phage MS2	5	Simulated solar	30 min	17

Summarily, a perspective of using different semiconductor photocatalysts against COVID-19 could be a potential strategy to combat the ongoing COVID-19 crisis. However, research on photocatalytic prevention and inactivation of the SARS-CoV-2 virus is still in the infancy stage and requires substantial efforts for better outcomes. Based on the antiviral activities of different photocatalytic materials involving metal oxides (TiO_2_, WO_3_, CuO, *etc.*), metal-free photocatalysts (graphene, GO, rGO, and g-C_3_N_4_), and 2D semiconductor materials, they are being employed as coatings for masks and air filters, paints, and disinfectants to prevent community spread of the deadly virus.^148–150^ Additionally, owing to the photothermal effect of MXenes and targeted drug-carrier ability of MOFs/COFs, it is highly anticipated that these advanced materials can be smartly and intensively explored for the therapeutic treatment of COVID-19 disease in the future.

## 3. Significance and roadmap to sustainable solutions for pandemics

**Fig. 10:**
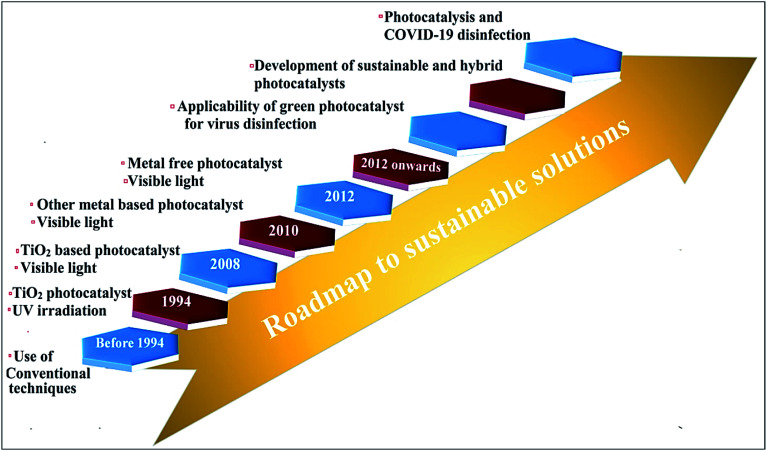
Roadmap depicting progression toward a sustainable solution for combating viruses through photocatalytic technology.

The impact of highly contagious COVID-19 on people and society is highly changeable and unthinkable, resulting in a high mortality rate in various countries.^147^ Thereby, keeping personal protection from rapid SARS-CoV-2 infection remains a serious and highly noteworthy challenging issue.^150^ Over the past few years, photocatalysis has been vastly explored for antiviral and antibacterial applications in living beings. The progression in photocatalysis in combating virus inactivation is depicted in Fig. 10. There are several measures including the use of PPE kits, social distancing, proper sanitization, surgical and medical (single use) masks that are suggested for COVID-19 control, and prevention for self-protection.^147–150^ Nevertheless, massive use of these preventive measures encounters significant challenges in the recycling and sterilizing of various utensils and cloths. The advantages of photocatalytic viral disinfection over homogeneous-phase advanced oxidation processes are well documented.

### 3.1 Designing rejuvenated, reusable, and biodegradable masks

To address various drawbacks associated with single useable masks, photocatalytic rejuvenation is a highly significant strategy that can sterilize the harmful microbes facially in the presence of light. Instead of using polypropylene as a precursor, alternative materials such as polyethylene oxide, cellulose nanofibers, and electrospinning of polyvinyl alcohol are also explored as effective materials for the fabrication of masks. As-fabricated sustainable and environmentally-friendly masks containing N–TiO_2_/TiO_2_ coating had 100% bacterial disinfection, indicating the efficiency of personal protective equipment (PPE) under ongoing and future pandemics.^148^ Photocatalytic self-sterilizing masks are designed for superior biodegradability, filterability, breathability, and mechanical strength that facilitate their handling and environmental impact.

### 3.2 Developing novel photoreactors

The spatial distribution of absorbed radiation in photocatalytic annular reactors is a crucial parameter for disinfecting harmful airborne microbes. Considering that, vast research efforts have been made to construct new photoreactors, such as packed-bed, monolithic, photocatalytic membrane, and micro-reactors to improve the efficiency and design of existing ones. In addition, the retention of airborne microbes in the bed, *i.e.*, filtration and the action of the generated ˙OH radicals in photoreactors are two main processes involved in photocatalytic disinfection. In a study, TiO_2_ coated glass rings with high filtration capacity, and low-pressure drops were filled inside a reactor that was irradiated by UV-A lamps both internally and externally. A 100% disinfection rate of bacterial spores in 1 h was observed inside the reactor, highlighting the excellent packing material employed in bio-aerosol deactivation to mitigate ongoing and future pandemics.^150^

### 3.3 Photocatalytic assisted microwave plasma-based pyrolyzer

Highly hazardous biomedical waste, including sharps, masks, gloves, bottles, pathological, chemical, radioactive, paper, *etc.* can be effectively treated with a simple, affordable, and highly efficient photocatalytic chemical route with high efficiency. Complete pyrolysis of typical wastes at high temperatures into liquid oil, gases, and char using an explorable microwave plasma-based pyrolyzer is a highly sustainable approach.^147^ Typically, pyrolysis of polystyrene plastic gives styrene, toluene like essential oils as reported in the literature survey, describing the efficiency of this process. Furthermore, it is a high energy-saving process as char obtained after pyrolysis can be effectively utilized as solid fuels, for generating carbon nanofilaments. Other different catalysts with high surface areas and generated pyrolytic oils with organic, inorganic compounds have much use as combustion fuels, transportation fuels, power generation, wood preservatives, *etc.*^147,148^

### 3.4 Use of photocatalytic air filters

It is of immediate importance to develop a potent prevention tool to reduce rapid transmission along with an improved inactivation rate of harmful pathogens. Thus, disposable face masks neither inactivate microbes nor block their entry from natural surroundings efficiently. The development of photoactive TiO_2_ nanowires-based reusable air filters with 100–200 m^2^ filter capacity is a promising sustainable solution reported for the first time. As-synthesized filters are much more effective owing to their high surface area, polycrystalline counterparts, and super-hydrophilicity. Generated ˙OH, HO_2_˙, H_2_O_2_, ^1^O_2_, ˙O_2_^−^ on the nanoporous TiO_2_ surface sustainably disinfect airborne bacteria, viruses including CoV-2. Moreover, as-fabricated filters further can also be used for purification or as conditioners.^149,150^ Thus, these above-mentioned sustainable, reusable, and eco-friendly methods could be a great alternative solution in dealing with the ongoing COVID-19 contagion.

## 4. Conclusions and future outlook

In summary, the global COVID-19 pandemic emergency has necessitated an urgent need for the development of a smart and efficient way to combat the coronavirus worldwide. Among various strategies, photocatalysis has been reported for the synergistic improvement of prevention, detection, diagnosis, and treatment of COVID-19 by using different types of catalysts under light illumination. As a green approach, solar light-driven photocatalysts have already been explored in the prevention and disinfection of various viruses and may ignite a ‘fresh start’ for the inactivation of SARS-CoV-2 with no issues such as stability, toxicity, cost-effectiveness, and availability. Considering that, in this review article, the existing progress of photocatalytic virucidal activities has been discussed and studied to present the latest advancements in fighting against the COVID-19 virus in an environmentally friendly way. Herein, the green aspects of photocatalysis have been explored along with its significance with respect to different photocatalysts including metal-oxide, metal-free, and other 2D materials for antiviral activities. Studies concerning the prevention and disinfection of distinct viruses and a promising path toward sustainable solutions for future pandemics have been pondered upon. Despite considerable research on photocatalytic viral inactivation, certain bottlenecks associated with semiconductor materials and process limits their photoactivity, which must be overcome in order to employ photocatalysis for SARS-CoV-2 disinfection in the future. Consequently, potential challenges in this field with a plausible future outlook are presented below:

• The recovery and the reusability of the suspended photocatalysts from the reaction solution are quite challenging owing to adsorbed species at the surface of the photocatalyst, which may or may not be degraded completely. Typically, the disinfection process *via* photocatalysis can be unsafe if the inactivation process is not completed and the photocatalyst still holds harmful viral species at the surface of the catalyst. Therefore, it is important to subject the photocatalyst under light irradiation after complete recovery from the reaction mixture in order to ensure the complete inactivation of any adsorbed viral species.

• In the case of photocatalytic coatings and membrane filters, the concept of self-sterilization is very crucial for long-run applicability and decline in critical waste generated *via* the disposal. However, there are very less reports which explain various strategies to improve the self-sterilization of photocatalytic coatings and enhance their reusability. Therefore, more research efforts are highly desirable to develop facile strategies, which promote the prevention, disinfection and reusability of photocatalytic coating materials.

• For waste water disinfection, the aggregation of nanosized photocatalytic material results in less active surface sites hence reduced photoactivity. Notably, immobilizing a photocatalytic material onto the porous or floating substrate can improve recovery along with agglomeration issues leading to enhanced reusability.

To conclude, for photocatalytic water disinfection, the barriers, which hinder long-scale applicability must be considered on priority bases for better results. Consequently, the complete eradication of the deadly SARS-CoV-2 by semiconductor-based photocatalysis demands much more extensive research.

## Conflicts of interest

There are no conflicts to declare.

## Supplementary Material
